# Concurrent cranial and cervical spine injuries by associated injury mechanisms in traumatic brain injury patients

**DOI:** 10.4102/sajr.v26i1.2321

**Published:** 2022-03-24

**Authors:** Pilasande Hlwatika, Timothy C. Hardcastle

**Affiliations:** 1 Department of Radiology, Inkosi Albert Luthuli Hospital, University of KwaZulu-Natal, Durban, South Africa; 2 Trauma Service and Trauma ICU, Inkosi Albert Luthuli Central Hospital, Durban, South Africa; 3 Department of Surgical Sciences, University of KwaZulu-Natal, Durban, South Africa

**Keywords:** concurrent cranial injury, cervical spine injury, post-traumatic coma, radiation, computed tomography scan

## Abstract

**Background:**

The incidence of concurrent traumatic brain injury (TBI) and cervical spine injury (c-spine) is relatively high, with a variety of risk factors.

**Objectives:**

The purpose of this study was to determine the incidence and related factors associated with combined cranial and c-spine injury in TBI patients by assessing their demographics and clinical profiles.

**Method:**

A retrospective study of patients attending the Trauma Centre at the Inkosi Albert Luthuli Hospital as post head trauma emergencies and their CT brain and c-spine imaging performed between January 2018 and December 2018.

**Results:**

A total of 236 patients met the criteria for the study; 30 (12.7%) patients presented with concurrent c-spine injury. Most TBI patients were males (75%) and accounted for 70% of the c-spine injured patients. The most common mechanism of injury with a relationship to c-spine injury was motor vehicle collisions (MVCs) and/or pedestrian vehicle collisions (70%). The risk factors associated with c-spine injury in TBI patients were cerebral contusions (40%), traumatic subarachnoid haematomas (36%) and skull fractures (33.3%). The statistically significant intracranial injury type more likely to have an associated c-spine injury was diffuse axonal injury (*p* = 0.04).

**Conclusion:**

The results suggest that concurrent TBI and c-spine injury should be considered in patients presenting with a contusion, traumatic subarachnoid haematoma and skull fracture. The high incidence of c-spinal injury and more than 1% incidence of spinal cord injury suggests that c-spine scanning should be employed as a routine for post MVC patients with cranial injury.

## Introduction

Trauma is one of the major causes of morbidity and mortality in children and young adults in South Africa (SA). According to a study by Norman et al., the injury-related mortality rate in SA is reported to be six times higher and road traffic injuries two times higher, compared with the global rate.^1^

Traumatic brain injuries (TBIs) are among the most common injuries that lead to hospitalisation, surgical interventions, permanent disabilities and, in severe cases, death.^2^ According to the United States of America’s Centre for Disease Control and Prevention, most TBIs are due to motor vehicle collisions (MVCs), sports and firearm-related injuries and other assaults.^2,3^ A study in Pietermaritzburg, KwaZulu-Natal (KZN), in 2014, reported that the most common cause associated with TBI was interpersonal violence (39.4%), with a male-to-female ratio of 4.7:1.^4,5^ Kong and Clarke analysed 5 years (2008–2011 and 2012–2014) of morbidity and mortality conferences (MMCs) performed in the Pietermaritzburg Metropolitan Trauma Service (PMTS) and reported that the highest percentage of patients were isolated TBI post-blunt trauma at 56.5%, with multiregional injuries in 21.3% thereof and isolated neck injuries at 1.5%.^6^

Milby et al. reported that there was a high prevalence of cervical spinal injury (c-spine) in TBI patients post-blunt trauma with 3.7% overall.^7^ A study by Fujii et al. estimated that the incidence of concurrent TBI and c-spine injury was between 1.7% and 8.0% in all trauma cases.^8^ Another study by Tian et al. found that patients who had TBI as a result of MVCs and a low Glasgow Coma Scale (GCS) score of less than or equal to 8 were associated with an increased risk of c-spine injury. The clinical evaluation of the TBI level using the GCS is scored as follows: mild (GCS: 13–15), moderate (GCS: 9–12) and severe (GCS: 3–8).^9^ According to Malale et al., the chance of an abnormal imaging finding increases with a decreasing GCS score.^10^ The other risk factors for concurrent c-spine injury and TBI included an older age group in MVCs, skull or facial fractures and chest, pelvic, upper limb or other spinal injuries.^8^ The incidence of concurrent head and c-spine injuries was documented as high as 9.3% by Thesleff et al. in an article published in 2017, with the retrospective data collected for cases between August 2010 and 2012.^11^ Failure to diagnose c-spine injury in a TBI patient can result in irreversible and devastating neurological damage. Therefore, the diagnosis of a c-spine injury in all patients presenting with TBI is an essential aspect of the trauma evaluation process.^7^

Using the general guideline, all patients presenting with a TBI and with an altered level of consciousness are treated as having a concurrent c-spine injury. Many c-spine injuries may be diagnosed utilising conventional cervical radiography. Although plain radiographs are relatively inexpensive and readily available in rural hospitals in SA, they are, however, associated with up to 15% missed injury rate compared with CT,^12^ which is currently considered as the gold standard for diagnosing a c-spine injury, with a better sensitivity of 98% compared with that of plain radiography at 54%.^13^

The high incidence of c-spine injury in patients with TBI led to CT evaluation of TBI and c-spine injuries in a government healthcare facility in Pietermaritzburg^13^ based on retrospective data collected from all the trauma patients from January 2016 to June 2016. Interestingly, the results showed that a combined diagnosis of TBI and c-spine injuries was relatively uncommon affecting 4.76% of the overall study population and was found mostly in patients involved in MVCs and pedestrian vehicle collisions (PVCs).

The aim of this study was to determine the incidence of and the associated risk factors for c-spine injuries in patients undergoing head scans for TBI, by describing the demographic and clinical profile of the patients, determining the predictive mechanism of injury, level of injury, severity of intracranial injuries and the site and nature of the cervical injuries in a Trauma Society of South Africa accredited level 1 trauma centre at Inkosi Albert Luthuli Central Hospital (IALCH) in Durban, KwaZulu-Natal, SA.

## Methods

This retrospective study was conducted between 01 January 2018 and 31 December 2018 at IALCH and included all patients attended to at the trauma centre as post head trauma emergencies. During this period, 272 patients had an initial CT brain and c-spine scan performed or, subsequent to the brain scan, had a CT cervical spine scan performed up to a week later. However, 36 of those patients were excluded from the study due to factors including lack of adequate history in their charts, no history of trauma and unavailability of images.

Data acquisition covered age, sex, mechanism of injury and level of consciousness (GCS) on arrival at the Trauma and Emergency Department; CT brain findings of acute traumatic intracranial abnormalities (primary and secondary injuries); CT cervical spine findings of trauma (fracture or subluxation) and the level of injury and extra-cranial injuries. The data were collected from the Picture Archiving and Communication System (PACS), Radiology Information System (RIS) and Hospital Information System (HIS) at IALCH. Images were re-reviewed by a single radiologist with more than 5 years of experience.

Descriptive statistics were used to summarise the data. Frequencies and percentages were used for categorical data such as the mode and severity of the injury (reported as median and interquartile range [IQR]). Frequency distributions of numerical data such as age were examined for normality and means (standard deviation [s.d.]) or medians (IQR). Group comparisons of risk factors among patients with and without c-spine injuries were tested with the chi-square or Fisher’s exact tests for categorical data and t-tests or Wilcoxon rank sum test for numerical data. A 5% significance level was used (*p* < 0.05).

### Ethical considerations

The study was registered as a sub-study of the existing research ethics approval by the UKZN Biomedical Research Ethics Committee (BCA207/09 – class approval for trauma and burns data at IALCH). The requirement for informed consent was waived because of the retrospective nature of the study. Patient anonymity was assured by the use of de-identified data.

## Results

The number of the patients who met the criteria for the study was 236. Of these, 30 (12.7%) presented with concurrent c-spine injury. The demographic and clinical profiles of all 236 TBI patients and the 30 patients with positive findings of a c-spine injury on a CT scan are summarised in Table 1. The predominant age group in both the total study population and in the c-spine injured patients was the 25- to 59-year age group and the majority were male. There were similar initial levels of consciousness of the total population versus the c-spine injured patients. Most patients had a severely decreased level of consciousness.

**TABLE 1:** Frequency distribution of age group, sex and level of consciousness in the total study population (*n* = 236) and the patients with a c-spine injury (*n* = 30).

Variable	All study cases		C-spine injuries	
	*n*	%	*n*	%
Total number of cases	236	100.0	30	12.7
**Age group**				
0–9	29	12.3	0	0.0
10–19	34	14.4	3	10.0
20–24	19	8.1	3	10.0
25–59	141	59.7	22	73.3
60–82	13	5.5	2	6.7
**Sex**				
Male	178	75.4	21	70.0
Female	58	24.6	9	30.0
**Level of consciousness (GCS)**				
Mild: 14–15	84	35.6	11	36.7
Moderate: 9–13	44	18.6	5	16.7
Severe: 3–8	98	41.5	12	40.0
Sedated	10	4.2	2	6.7

GCS, Glasgow Coma Scale.

Table 2 shows the different mechanisms of injury. The mechanism of injury associated with the highest number of admitted patients was MVC (including PVC), accounting for 66.1% (156/236). In patients with c-spine injuries, MVC accounted for 70.0% versus 65.5% in patients without a c-spine injury. There was no statistically significant association between MVC (including PVC) and a c-spine injury (*p* = 0.887).

**TABLE 2:** Mechanism of injury in patients with a c-spine injury versus patients with no c-spine injury.

Mechanism of injury	C-spine injury				*P*
	Yes (*n* = 30)		No (*n* = 206)		
	*n*	%	*n*	%	
All cases	30	12.7	206	87.3	-
**Mechanism**	-	-	-	-	0.89
MVC (including PVC)	21	70.0	135	65.5	-
Blunt assault	3	10.0	31	15.0	-
Fall	2	6.7	17	8.3	-
Penetrating injury/gun/stab	2	6.7	12	5.8	-
Object fell onto patient	2	6.7	4	1.9	-
Unknown	0	0.0	4	1.9	-
Other	0	0.0	3	1.5	-

MVC, motor vehicle collisions; PVC, pedestrian vehicle collisions.

All 30 of the c-spine injured patients had a cervical vertebral fracture, with 6 of the 30 (20%) having a concomitant subluxation. In terms of the level of injury, 11 cases had an injury in the upper cervical spine (base of the skull to the C2 level), 15 cases in the sub-axial c-spine (C3 to T1) and 4 cases in both the upper cervical spine and sub-axial spine. Nineteen (63.3%) of the patients with a c-spine injury had only a bone injury, while six (20.0%) also had a symptomatic spinal cord injury. This was equal to the overall incidence of the symptomatic spinal cord injury of 6 of 236 (2.54%). There was insufficient information in the charts of 16.7% (5/30) to determine the presence of a cord injury. The only statistically significant intracranial injury type more likely to have an associated c-spine injury was diffuse axonal injury (*p* = 0.04). The other intracranial injury types, as depicted in Table 3, which were more common than diffuse axonal injury in terms of percentage, were similarly equally common in patients without c-spine injury. The extra-cranial injuries commonly found in the patients with a positive c-spine injury included chest injury (56.7%), lower limb injury (30.0%) and facial injury (26.7%).

**TABLE 3:** Intracranial injury types associated with a c-spine injury.

Intracranial injury type	Cervical spine injuries (c-spine injuries)				*p*
	Yes (*n* = 30)		No (*n* = 206)		
	*n*	%	*n*	%	
**Skull fracture**					
Yes	10	33.3	92	44.7	0.24
No	20	66.7	114	55.3	-
**Extradural haematoma**					
Yes	5	16.7	22	10.7	0.34
No	25	83.3	184	89.3	-
**Subdural haematoma**					
Yes	6	20.0	65	31.6	0.20
No	24	80.0	141	68.4	-
**Traumatic subarachnoid haematoma**					
Yes	11	36.7	70	34.0	0.84
No	19	63.3	136	66.0	-
**Pneumocephalus**					
Yes	3	10.0	29	14.1	0.78
No	27	90.0	177	85.9	-
**Cerebral contusions (haemorrhagic or non-haemorrhagic)**					
Yes	12	40.0	110	53.4	0.17
No	18	60.0	96	46.6	-
**Diffuse axonal injury**					
Yes	8	26.7	26	12.6	0.04
No	22	73.3	180	87.4	-
**Cerebral oedema**					
Yes	5	16.7	43	20.9	0.59
No	25	83.3	163	79.1	-
**Compression of the cerebrospinal fluid spaces**					
Yes	9	30.0	51	24.8	0.54
No	21	70.0	155	75.2	-
**Acquired intracranial herniation**					
Yes	5	16.7	37	18.0	0.83
No	25	83.3	169	82.0	-

## Discussion

The demographic profile of the patient group indicated that males accounted for over 75% of the group. Males generally constitute a higher percentage of those exposed to intracranial injuries.^14^ Young men may often be involved in vehicle-related incidents, substance abuse and interpersonal violence leading to TBIs.

Several modes of injury have been implicated in concurrent cranial and c-spine injury patients, including MVCs, PVCs, assaults, falls and gunshot wounds.^15^ In a study conducted by Hardcastle et al. in 2013, the authors report that interpersonal violence and MVCs constitute a major healthcare burden on the South African public healthcare system, constituting 18.0% of the emergency care burden.^16^ In this study, MVC (including PVC) and assault were the most common mechanism of injury (70.0%) followed by fall (8.1%) and penetrating/gun/stab wounds (5.9%).

According to Frye et al. in 2002, intracranial haemorrhage is a common finding in moderate and severe head injuries, but it is not a predictor of occult c-spine injury.^17^ In this study, most patients presented with cerebral contusions (51.7%) and skull fractures (43.2%). Hills and Deanne reported the GCS scores in head injuries associated with a c-spine injury. Traumatic brain injury patients with GCS scores of 8 or less were at a higher risk of spine injuries.^18^ Similarly, this study confirmed that 41.5% of patients who presented with a severe GCS score of 8 had a greater chance of abnormal CT findings and/or were at the highest risk for c-spine injuries.

South Africa has a very high prevalence of trauma that necessitates many CT examinations. Although IALCH, located in Durban, KZN, provides level 1 trauma services, the province needs more hospital facilities that provide such specialised trauma care services. However, the increase in CT imaging may expose TBI patients to radiation and may increase the lifetime risk of cancer, which may lead to greater public health problems.^19,20,21,22,23,24^ To reduce the complications of radiation, the As Low As Reasonably Achievable (ALARA) principle must be applied to reduce radiation exposure.

The limitation of this retrospective chart review study was that the study was performed only in one setting at the regional neurosurgical centre (IACLH, KZN). Therefore, the results cannot be necessarily generalised to less severe injuries; however, it confirms that in cases with severe TBI, the c-spine should be imaged at the same time as the brain, as the incidence of c-spine injury and cord injury both exceed the ‘1%’ medicolegal risk rule.^25^ In the case of the CT imaging reports, the expertise or experience of the reporting radiologist could also affect the outcome of the results of the study, which was reduced by the image review performed by the lead investigator.

## Conclusion

The early diagnosis of both a TBI and c-spine injury is essential to manage the c-spine injury and minimise adverse events. Poor management of c-spine injuries may result in irreversible and devastating neurological damage. Motor vehicle collisions and TBI patients with GCS scores of 8 or less and specific intracranial injuries have a greater chance of abnormal CT findings and are at high risk for c-spine injuries. Given the high incidence of c-spinal injuries and the more than 1% incidence of spinal cord injury, combined brain and c-spine scanning should be performed routinely in MVC and PVC.
